# Modulation of the Disordered Conformational Ensembles of the p53 Transactivation Domain by Cancer-Associated Mutations

**DOI:** 10.1371/journal.pcbi.1004247

**Published:** 2015-04-21

**Authors:** Debabani Ganguly, Jianhan Chen

**Affiliations:** 1 Department of Biochemistry and Molecular Biophysics, Kansas State University, Manhattan, Kansas, United States of America; 2 Indian Institute of Engineering Science and Technology, Shibpur Howrah, India; Baltimore, UNITED STATES

## Abstract

Intrinsically disordered proteins (IDPs) are frequently associated with human diseases such as cancers, and about one-fourth of disease-associated missense mutations have been mapped into predicted disordered regions. Understanding how these mutations affect the structure-function relationship of IDPs is a formidable task that requires detailed characterization of the disordered conformational ensembles. Implicit solvent coupled with enhanced sampling has been proposed to provide a balance between accuracy and efficiency necessary for systematic and comparative assessments of the effects of mutations as well as post-translational modifications on IDP structure and interaction. Here, we utilize a recently developed replica exchange with guided annealing enhanced sampling technique to calculate well-converged atomistic conformational ensembles of the intrinsically disordered transactivation domain (TAD) of tumor suppressor p53 and several cancer-associated mutants in implicit solvent. The simulations are critically assessed by quantitative comparisons with several types of experimental data that provide structural information on both secondary and tertiary levels. The results show that the calculated ensembles reproduce local structural features of wild-type p53-TAD and the effects of K24N mutation quantitatively. On the tertiary level, the simulated ensembles are overly compact, even though they appear to recapitulate the overall features of transient long-range contacts qualitatively. A key finding is that, while p53-TAD and its cancer mutants sample a similar set of conformational states, cancer mutants could introduce both local and long-range structural modulations to potentially perturb the balance of p53 binding to various regulatory proteins and further alter how this balance is regulated by multisite phosphorylation of p53-TAD. The current study clearly demonstrates the promise of atomistic simulations for detailed characterization of IDP conformations, and at the same time reveals important limitations in the current implicit solvent protein force field that must be sufficiently addressed for reliable description of long-range structural features of the disordered ensembles.

## Author Summary

Tumor suppressor p53 is the most frequently mutated protein in human cancers. Clinical studies have suggested that the type of p53 mutation can be linked to cancer prognosis, response to drug treatment, and patient survival. It is thus crucial to understand the molecular basis of p53 inactivation by various types of mutations, so as to understand the biological outcomes and assess potential cancer intervention strategies. Here, we utilize a recently developed replica exchange with guided annealing enhanced sampling technique to calculate well-converged atomistic conformational ensembles of the intrinsically disordered transactivation domain (TAD) of tumor suppressor p53 and several cancer-associated mutants in an implicit solvent protein force field. The calculated ensembles are in quantitative agreement with several types of existing NMR data on the wild-type protein and the K24N mutant. The results suggest that, while all sequences sample a similar set of conformational substates, cancer mutants could introduce both local and long-range structural modulations and in turn perturb the balance of p53 binding to various regulatory proteins and further alter how this balance is regulated by multisite phosphorylation of p53-TAD. The study also reveals important limitations in implicit solvent for simulations of disordered proteins like p53-TAD.

## Introduction

Cellular signaling and regulation are frequently mediated by proteins that, in part or as a whole, lack stable structures under physiological conditions [1–3]. Such intrinsically disordered proteins (IDPs) are over-represented in disease pathways [4,5]. About ~25% of disease- associated missense mutations can be mapped into predicted disordered regions [6] (although cancer mutations appear to prefer ordered regions [7]). Many disease mutations in disordered regions have been predicted to alter the residual structure level [8], which could potentially perturb interaction networks and lead to mis-signaling and mis-regulation. Establishing the biophysical basis of how disease mutants affect the “structure”-function relationship of IDPs is a formidable task. It requires detailed characterization of the disordered conformational ensembles, which are not amenable to traditional structural determination using either X-ray crystallography or nuclear magnetic resonance (NMR) spectroscopy [9–11]. For disordered protein states, only ensemble-averaged properties are generally measured [12,13], and single-molecule techniques are often limited by low spatial resolution and labeling complications [14–16]. Recovering the underlying structural heterogeneity using ensemble-averaged properties is fundamentally underdetermined; there is not sufficient constraint (or information) to uniquely define the structure ensemble based on averaged properties alone. A possible strategy to overcome this fundamental limitation is to leverage significant recent advances in physics-based protein force fields and enhanced sampling techniques to calculate *de novo* structural ensembles [10,17]. Structural data from NMR and other biophysical experiments can be then used for independent validation, but not as structural restraints during the ensemble calculation. This strategy has proven effective enough to provide useful insights on studies of several relatively small IDPs [18–23]. An important caveat is, however, *de novo* ensembles will inevitably contain artifacts due to persisting limitations in the current protein force fields as well as conformational sampling capability. Nonetheless, certain systematic artifacts could be suppressed by examining how the calculated ensembles depend on sequence variations, post-translational modifications, and/or solution conditions [23,24].

To assess the efficacy of atomistic simulations for understanding the mutant-structure-function relationship of IDPs, we exploit the intrinsically disordered transactivation domain (TAD) of the tumor suppressor p53 and its cancer-associated mutations as a model system of great biological and biomedical significance. p53 is the most frequently mutated protein in cancer [25,26]. The p53 levels are kept low in unstressed cells through continuous proteasomal degradation. Cellular stresses such as DNA damage, initiate a cascade of phosphorylation events that stabilize and activate the p53 protein [27]. Accumulation of activated p53 induces the transcription of genes involved in cell cycle arrest and apoptosis, thus suppressing cell transformation and tumor formation [28]. Most human cancers exhibit defects in the p53-signaling pathway, over 50% of which involve inactivated p53 due to various mutations [29,30]. Clinical studies of breast cancer have indicated that the type of p53 mutation can be linked to cancer prognosis and response to drug [31]. It is thus crucial to determine the molecular basis of p53 inactivation by various types of mutations, so as to understand the biological consequences and predict potential treatment responses and patient survival.

**Fig 1:**
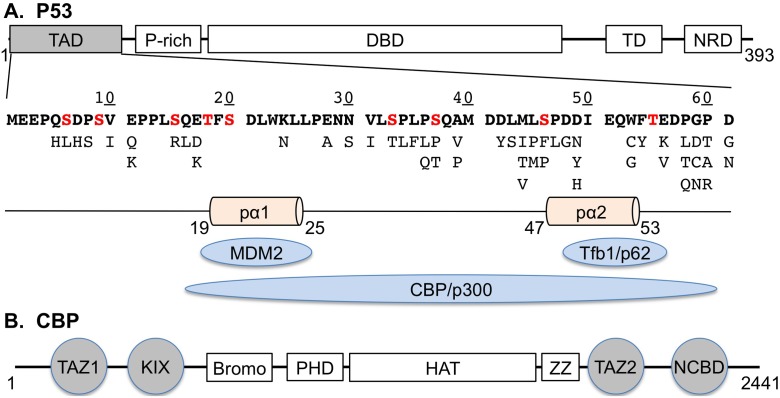
Domain structures of A) p53, and B) CREB-binding protein (CBP). Also shown in A) include: the sequence of p53-TAD (in bold fonts with phosphorylation sites marked in red), known cancer mutants (in light fonts below the sequence; three additional mutants, P60L/S/Q, are not shown), and its key interaction partners to be studied. Both TAD sub-domains, TAD1 (1–40) and TAD2 (41–61), contain short helices (pα1, pα2) that form upon specific binding to various targets. Four separate CBP domains (colored in grey) can interact with p53-TAD. *Abbreviations*: TAD: transactivation domain (1–61); P-rich: proline rich region; DBD: DNA-binding domain (102–292); TD: tetramerization domain (325–356); NRD: negative regulatory domain; TAZ1 (340–439) & TAZ2 (1764–1855): cysteine-histidine-rich regions; KIX (586–572), NCBD: nuclear receptor coactivator binding domain (2059–2117).

The p53 protein contains several distinct functional domains (Fig 1A). The core DBD domain binds to the regulatory regions of target genes, and the terminal domains interact with many proteins that together tightly regulate the p53 protein level, localization, oligomerization and activity [26]. The primary focus of existing structural and functional studies has been on cancer mutants in DBD [32], which harbors over 80% of p53 cancer mutations including established cancer “hot spots” [33]. Aided by several crystal structures [34,35], the molecular basis for p53 inactivation of DBD cancer mutants can be understood in terms of either disrupting DNA contacts, perturbing the structure of DNA-binding interface, or affecting the DBD stability [32]. In contrast, very little is known about the structural and functional impacts of cancer mutants in the regulatory domains and particularly TAD. This could be attributed to much lower prevalence, and thus perceived importance, of cancer mutants outside of DBD (e.g., ~1 per residue in TAD vs. ~6 per residue in DBD) [33]. Nonetheless, TAD cancer mutants appear to be frequently associated with some cancers. Two out of the thee documented female genital cancers contain mutants in TAD (E17D and K24N); over 5% nasal cavity, tonsil, salivary gland and parotid gland cancers involve mutated TAD (statistics extracted from the IRAC TP53 mutation database, version R15 [33]). At present, available functional knowledge of all known TAD cancer mutants (see Fig 1A) largely comes from a single yeast-based transcriptional activity essay study of all possible point mutations in the entire coding region of p53 gene [36] (with a few exceptions [30,33]). Moreover, no structural or molecular interaction data is available on any TAD cancer mutants except K24N [37].

A key complication in molecular studies of p53 TAD cancer mutants is that, in contrast to DBD with a stable fold, TAD is an IDP and must be described by heterogeneous structure ensembles [38–43]. In this work, we exploit the recently developed replica exchange with guided annealing (RE-GA) enhanced sampling technique [44,45] to calculate disordered ensembles of p53-TAD at atomistic level and examine how cancer-associated mutations could modulate the disordered ensembles to potentially disturb p53’s interactions with key regulatory proteins. RE-GA extends the popular temperature replica exchange (T-RE) method by introducing annealing cycles, during which the temperature exchange attempt patterns are modified for a selected replica to guide its diffusion through the temperature ladder more rapidly. The GA cycles help to overcome the limitation of RE in accelerating entropically limited cooperative conformational transitions [46–48], albeit at the expense of compromising the detailed balance for systems with large activation enthalpies [44]. For IDPs with relatively small conformational transition barriers, numerical experiments and atomistic simulations of a small 28-residue IDP have demonstrated that RE-GA introduces minimal conformational biases and could generate converged ensembles with 3–5 fold speedup compared to T-RE [44]. The convergence of RE-GA simulations will be carefully examined by comparing results from independent simulations initiated from contrasting structures. The quality of simulated ensembles will be critically assessed by direct comparison with a wide range of existing data that provide structural information on both the secondary and tertiary levels for the wild-type protein and one of its mutants [37,39,40,43]. Further analysis of all resulting atomistic ensembles will then be performed to obtain a preliminary understanding of how cancer-associated mutations may introduce both local and long-range structural changes in unbound p53-TAD, which could have functional consequences on how p53-TAD may differentially interact with key regulatory pathways and on how these differential interactions may be regulated through multi-site phosphorylations.

## Results

### Convergence of the simulated ensembles

**Fig 2:**
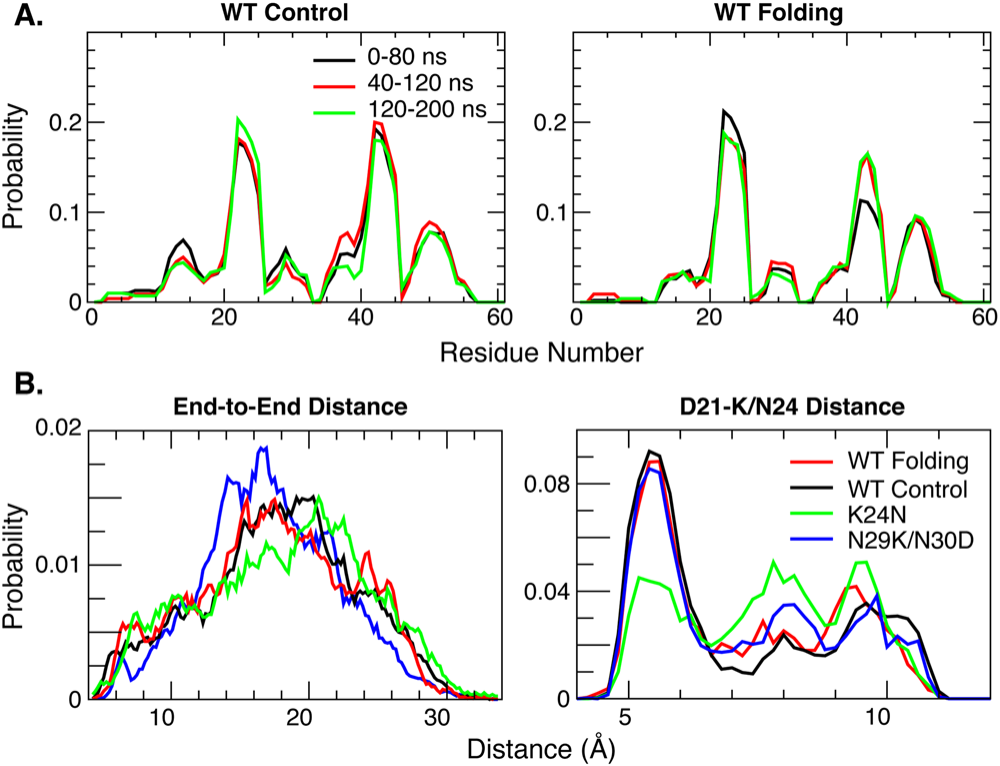
A) Averaged residue helicity profiles calculated using different 80-ns segments of the control and folding RE-GA simulations of wild-type p53-TAD. B) Probability distributions between termini and D21-K/N24 calculated from the last 80-ns of RE-GA simulations of the wild-type p53-TAD and two cancer-associated mutants. The inter-residue distances were calculated as the distances between corresponding CA atoms.

**Fig 3:**
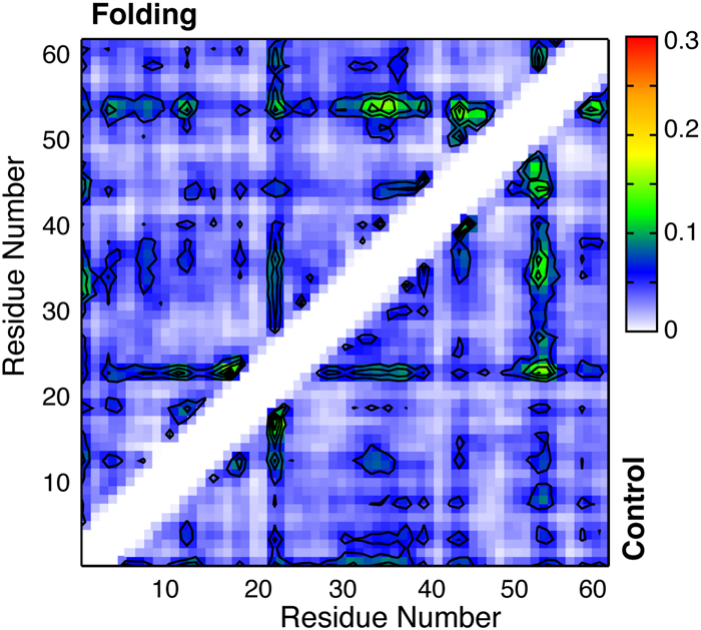
Probabilities of long-range contacts calculated from the last 80-ns segments of the folding (upper half) and control (lower half) RE-GA simulations. Contours are drawn from 0.06 with an equidistant increment of 0.04. Residues are considered to be in contact if the minimal heavy atom distance is no greater than 4.2 Å. The correlation efficient of two contacts is 0.91 and the RMSD is 0.016.

The convergence of the simulated ensembles has been evaluated by examining the self-convergence of various one-dimensional and multi-dimensional distributions, and more critically by comparing the results derived from independent control and folding runs that were initiated from contrasting initial structures. As shown in Fig 2 for the wild-type p53-TAD, the residue helicity profiles calculated using various 80-ns segments quickly reach stationary states, showing very small differences between profiles calculated using data from 40–120 ns or 120–200 ns of the simulations (Fig 2A). The simulated ensembles for all five p53-TAD cancer mutants display similar convergence behaviors (see S1 Fig). Importantly, the profiles calculated using the last 80-ns segments of the control and folding runs agree very well, with an overall RMSD of 0.014. Similar observations can be made on comparing various distributions of 1D residue-residue distances (e.g., Fig 2B, red and black traces). The simulated ensembles also appear to converge well on level of two—dimensional distributions, which is very difficult to achieve for IDPs of the size of p53-TAD. S2 Fig illustrates that helical substate distributions largely stabilize by the end of 200-ns RE-GA simulations for both the wild-type p53-TAD and its cancer mutants and that the final distributions from the control and folding runs are largely consistent. Furthermore, as shown in Fig 3, the structural ensembles derived from the control and folding simulations of the wild-type protein contain essentially identical sets of long-range contacts and with largely similar probabilities. The correlation coefficient of the two contact maps is 0.91 and the RMSD is 0.016. The level of convergence observed here for local and long-range structural properties of a 61-residue IDP is noteworthy. It provides a solid basis for detecting potentially subtle structural impacts of cancer-associated mutations.

### Comparison with NMR: Local structural propensities and long-range ordering

**Fig 4:**
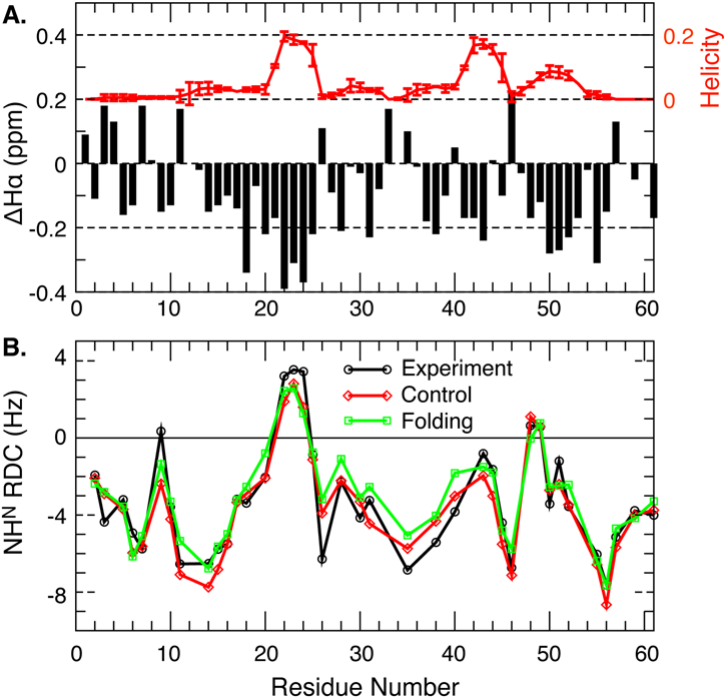
A) Comparison of the average residue helicity profile with the secondary Hα chemical shifts for the wild-type p53-TAD[38]. The uncertainties of the average residue helicities were estimated as the difference between values calculated from the folding and control RE-GA runs (see Fig 2A). The reference random coil values were taken from statistics of the BMRB database[50]. B) Back-calculated RDC profiles in comparison with the experimental one[39]. Note that the calculated profiles were globally scaled to best reproduce the experimental values.

The quality of the simulated ensembles has been assessed by comparing to existing experimental data that provide structural information on both the secondary and tertiary levels [37–40,43]. As shown in Fig 4A, the simulated helicity profile for the wild-type p53-TAD is highly consistent with NMR secondary chemical shift and NOE analysis[38], predicting three partial helices in the same regions that show significant negative secondary chemical shifts, namely residues 18–27, 40–44 and 48–52. These are also the same regions where short helices have been observed when p53-TAD is bound to various targets (see Fig 1A). The partial helices spanning residues 40–44 and 48–52 have been generally classified as turns I and II in previous NMR studies [38]. Nonetheless, continual sequential *d*_NN_ NOEs have been detected in both regions, which support the presence of residual helices [38]. The most recent NMR analysis has estimated that the average helicity in residues 17–29 is about 11.2% [37], which is quantitative agreement with the calculated value of ~10±1% in residues 18–27 from the simulations. Furthermore, as shown in Fig 4B, the theoretical RDC profiles derived from the simulated ensembles agree very well with the experimental one measured at 800 MHz [39]. For disordered protein states, RDC has been shown to be mainly sensitive to local secondary structures, particularly partial helices [39,49]. The agreement between calculated and measured RDC profiles thus further supports the notion that local structural properties of the simulated ensembles are most likely realistic.

**Fig 5:**
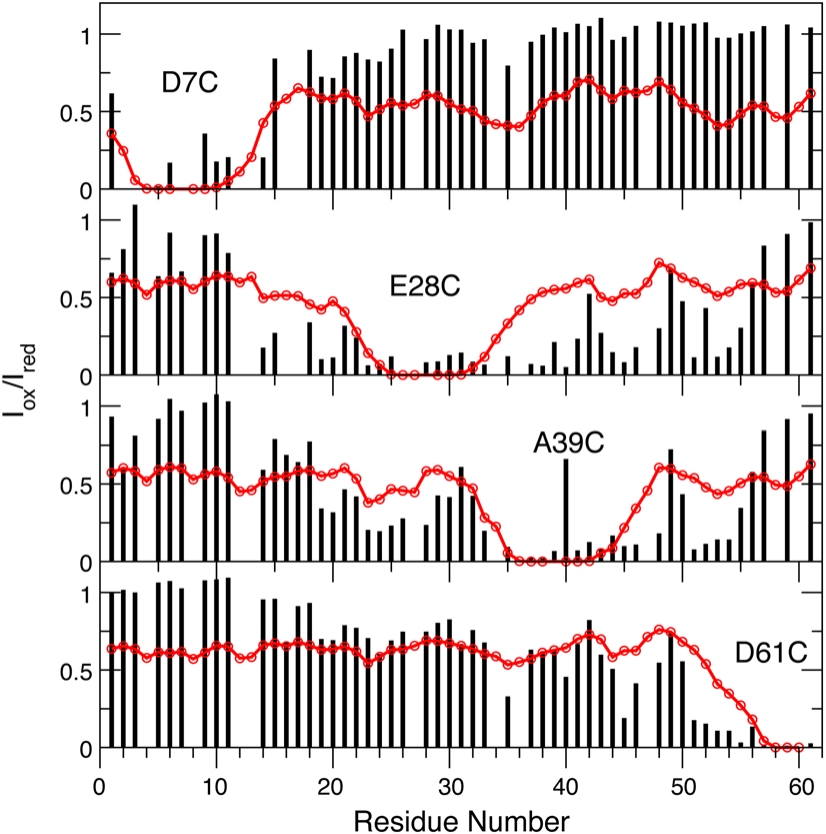
Comparison of theoretical (red traces) and experimental (black bars) PRE effects induced by four site-specific spin labels. The theoretical profiles were calculated from the last 80-ns of the folding simulation of wild-type p53-TAD. The experimental profiles were extracted from the Supplementary Materials of Stancik et al. (2008) [43]. The correlation coefficient between the theoretical and experimental profiles is 0.5.

Long-range tertiary structural properties of the simulated ensembles have been examined based on their ability to reproduce the experimental PRE effects [42]. PRE coupled with site-directed spin-labeling techniques is one of the most powerful techniques for characterizing transient long-range contacts of disordered proteins [51–53]. Relaxation enhancement of a given proton depends sensitively on its distance from the unpaired electron of the paramagnetic spin label, with an *r* ^-6^ dependence. PRE is thus uniquely suitable for detecting weakly populated transient contacts. At the same time, dominated by contributions from compact conformers, PRE is insensitive to members of the ensemble with large electron-nuclear distances. This property renders it generally unfeasible to calculate reliable structural ensembles for disordered protein states based on the PRE distances alone [54]. Nonetheless, the ability of PRE experiments to provide ensemble-averaged distance information between site-specific spin labels and *all* protons in the protein is extremely valuable for global validation of atomistic ensembles from *de novo* simulations. Fig 5 compares the theoretical PRE profiles calculated from the last 80-ns of the folding RE-GA simulation of wild-type p53-TAD with experimental results previously measured for four site-specific spin labels[43]. A key observation is that the theoretical profiles do not reach the 1.0 (no broadening) limit in any case. This suggests that the atomistic ensemble is overly compact, likely due to the known tendency of the GBSW/SA implicit force field to over-stabilize intra-peptide interactions [55,56]. Indeed, the end-to-end distance of the simulated ensemble (Fig 2B) appears substantially under-estimated compared to the single molecule FRET data [40]. Nonetheless, the calculated PRE profiles display fine features that appear to resemble the experimental ones. The simulations predict stronger PRE broadening in similar regions detected experimentally for all four spin-labeling sites located strategically to cover the whole sequence. The overall correlation coefficient of the theoretical and experimental PRE effects is about 0.5, which is far from ideal but nonetheless meaningful. The implication is that, despite clear over-compaction, transient long-range contacts in the simulated ensembles are likely genuine, albeit likely with systematically elevated or skewed probabilities.

We note that it is highly nontrivial for *de novo* atomistic simulations to generate well-converged ensembles for a 61-residue IDP like p53-TAD with non-trivial structures and achieve a high level of agreement with NMR on both secondary and long-range structural features. Implicit treatment of solvent environment is critical to reduce the computational cost, as also demonstrated quite extensively for other long IDPs [57–60]. The tendency of GBSW/SA to over stabilize collapsed structures, however, has hindered the ability of traditional T-RE simulations to generated converged ensembles for long IDPs, requiring us to adopt the RE-GA enhanced sampling here. With compromised detailed balance due to the GA cycles, the probabilities of high energy states tend to be over estimated when separated by large energy barriers [44]. Taken together, long-range structure features predicted by the current simulations should be considered qualitative at best. We note that several recently developed enhance sampling techniques may allow one to overcome the sampling limitation without compromising the detailed balance [61,62]. It should also be emphasized that agreement on average properties itself as discussed above does not establish the reliability of the whole ensemble, due to the under-determined nature of calculating heterogeneous structure ensembles. An essential validation will be the atomistic simulation’s ability to recapitulate the affects of mutations or post-translational modifications on the conformational properties. As will be discussed below, the latter appears to be the case for the K24N mutant.

### Mutant modulation of p53-TAD local and long-range conformations

**Fig 6:**
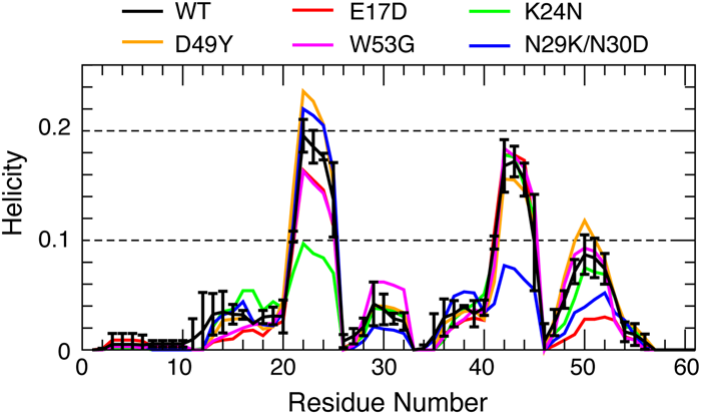
Residue helicity profiles for the wild-type p53-TAD and five cancer mutants, derived from the last 80-ns segments of the RE-GA simulations. Estimated uncertainties are similar for all profiles and only shown for the wild-type for clarity.

**Fig 7:**
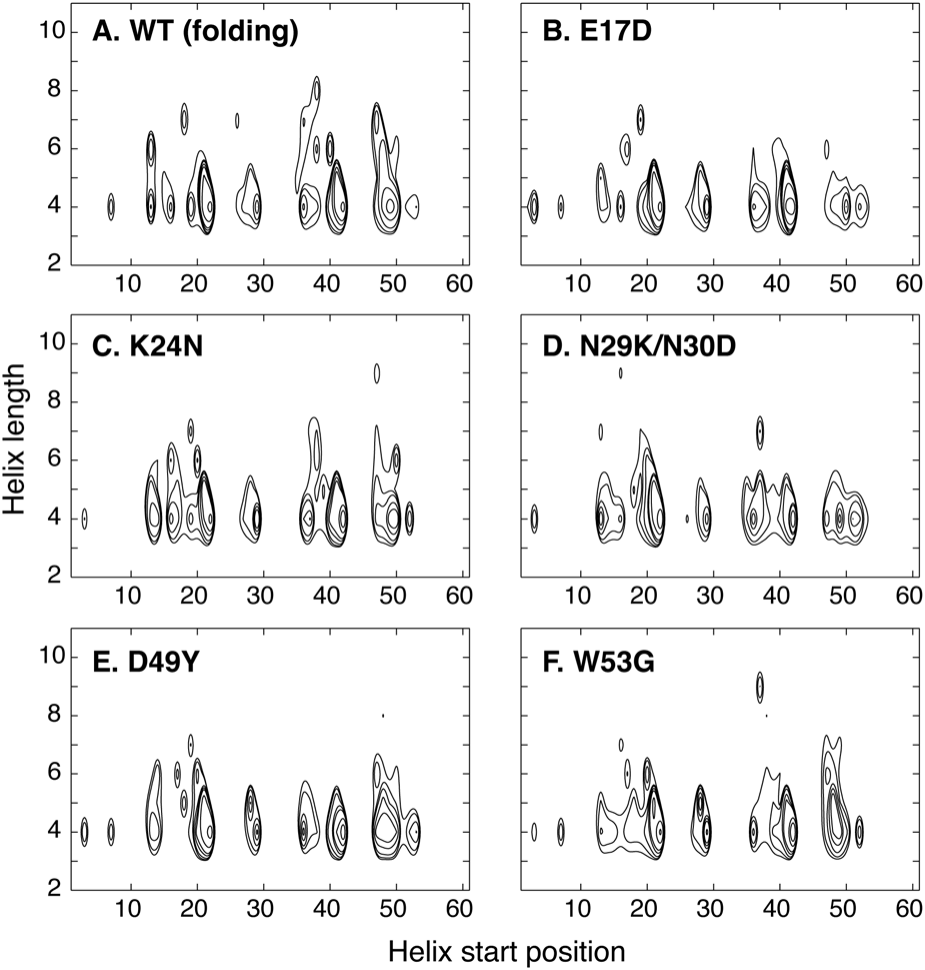
Distributions of helical substates of the wild-type p53-TAD and five cancer mutants, calculated from the last 80-ns of folding RE-GA simulations. Contours are drawn 0.001, 0.002, 0.004, 0.008, 0.012, 0.024 and 0.048.

**Fig 8:**
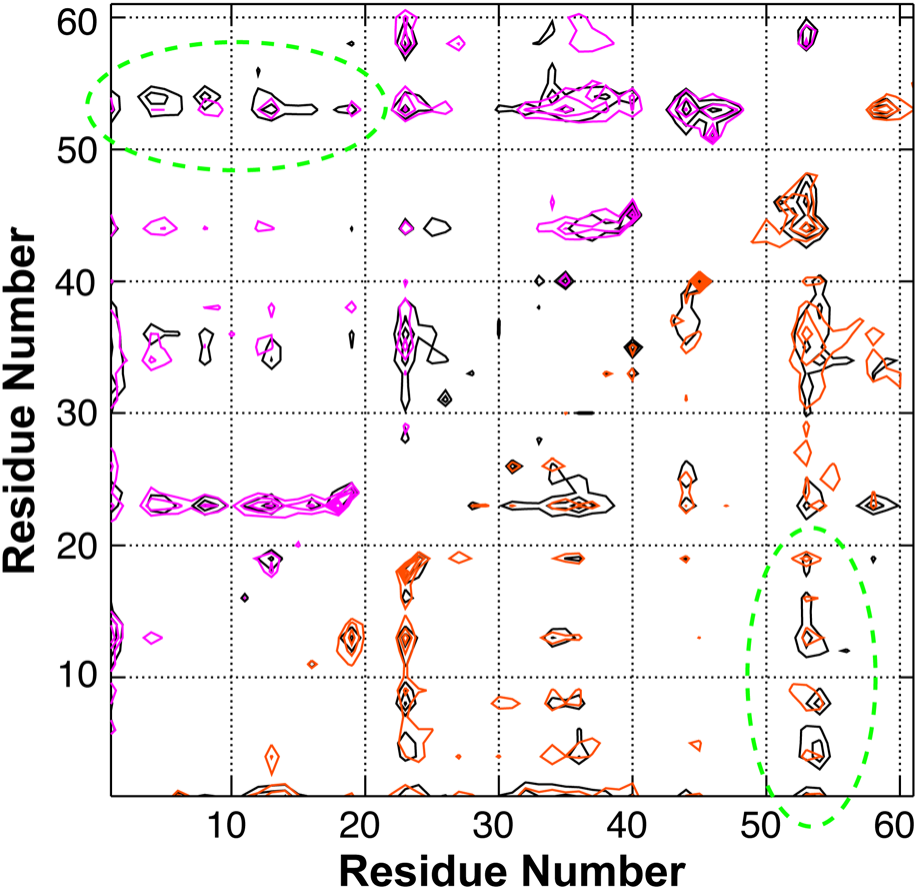
Average contact probabilities derived from RE-GA simulations of the wild-type p53-TAD (black contours), K24N (red; lower half), and N29K/N30D (violet; upper half).

In Fig 6, we first examine the effects of cancer-associated mutations on residue helical propensities of p53-TAD. Clearly, all cancer-associated mutants contain residual helices in the same regions as observed for the wild-type protein. However, the mutations appear to frequently modulate average helical propensities. Most effects are local. For example, the largest effects of replacing Trp53 with the helix breaking Gly residue are observed near residue 53, where the peak residue helicity is reduced from ~8% (black trace) to ~3% (purple trace). K24N mutation mainly reduces the helicity in residues 18–27, from an average of ~10% to ~5%. We note that the predicted helicity reduction of K24N is in quantitative agreement with NMR secondary chemical shift analysis [37]. The effect of K24N mutation may be attributed to direct disruption of the Asp21-Lys24 salt bridge, which has been suggested to stabilize the local partial helices [38]. As shown in Fig 2B, the probability of forming contacts between residues 21 and 24 is ~50% lower for K24N mutant than the wild type (green trace). On the helical substate level, while all p53-TAD sequences simulated here apparently sample a similar, if not identical, set of partial helices (Fig 7), their occupancies appear to be sensitive to mutations. We note that the convergence of helical substate distributions is more limited compared to average residue helicity profiles (S2 Fig). Nonetheless, the level of redistribution of among helical sub-states due to mutation appears significant. In particular, the differences between distributions calculated from folding and control RE-GA simulations of the wild-type sequence are considerably smaller than those between the wild-type and mutant distributions (S2 Fig). On the tertiary level, all sequences are extremely heterogeneous. Clustering analysis with 5 Å Cα RMSD cutoff leads to numerous small clusters for all ensembles, with very few clusters occupied over 1% (see S3–S8 Figs). Using larger cutoff values reduces the total number of clusters identified but no dominant clusters would emerge. Interestingly, on average all p53-TAD constructs simulated here appear to sample similar sets of long-range residue-residue contacts, even though cancer mutants do clearly impact their prevalence in the disordered ensemble (see Fig 8).

Intriguingly, several cancer mutants are predicted to lead to helicity changes in regions sequentially distal from the mutation sites. For example, besides significantly reducing the local helical propensity, W53G also leads a slight decrease in helicity within residues 18–27 (Fig 6, purple trace). The most striking case is N29K/N30D, which reduces average residue helicities in the distal regions of residues 40–44 and 48–52 by ~50%, but has minimal impact in the local region of residues 18–27 (blue trace). This is a potentially important observation, and suggests that long-range coupling exists among various residual structures of p53-TAD. The predicted long-range coupling is not likely an artifact of over compaction due to limitations of GBSW/SA. Similar long-range coupling in p53-TAD dynamics has been detected in a recent florescence quenching study of p53-TAD [41]. The existence of transient long-range contacts between residual helices is also evident in PRE experiments[43]. As shown in Fig 5, paramagnetic spin labeling at E28C leads to strong broadening around residues 43–47 and 52–54. Conversely, labeling at A39C leads to significant broadening around residues 18–27. Interestingly, comparing the contact probability maps (Fig 8) suggests that both K24N and N29K/N30D appear to weaken long-range contacts between the N- and C-terminal segments compared to the wild type p53-TAD (Fig 8, circled areas). These segments of substantial helical propensities are responsible for p53’s specific interactions with numerous regulatory proteins (e.g., see Fig 1A). At present, little concrete biophysical data is available on how TAD cancer mutants may perturb p53’s interaction with various regulatory proteins. The only molecular data available is that K24N does not significantly affect MDM2 binding due to an apparent enthalpy-entropy compensation[37], but its impacts on binding to CBP domains are not known. It is plausible the resulting structural changes in the disordered ensembles could have impacts on molecular interactions of p53 as well as their post-translational regulation.

## Discussion

The p53 protein level and activity are tightly regulated through coordinated interactions of TAD with negative regulators MDM2 and MDMX (mouse double minute 2 and 4) and the general transcriptional coactivators CBP and p300 [63] (see Fig 1B). Unphosphorylated p53-TAD binds to MDM2 with sub-micromolar affinity, which promotes polyubiquitination and degradation of p53 through MDM2’s E3 ubiquitin ligase activity[64,65]. Recent NMR and calorimetry studies showed that multisite phosphorylation of TAD reduced binding to MDM2 (by up to 24X, or ΔΔG ~ −1.9 kcal/mol), and at the same time provided graded enhancement of binding to CBP/p300 domains (by up to 80X, or ΔΔG ~ +2.6 kcal/mol)[66–68]. These effects together dramatically shift the balance towards favoring binding to CBP/p300, up to 1000-fold. The graded dependence on the extent of p53 phosphorylation provides a mechanism for gradually increasing p53 response under prolonged genotoxic stress[69]. Nonetheless, precisely how phosphorylation regulates the binding affinities is not entirely clear. Phosphorylation may simply provide a new interaction site and/or disrupt the binding interface. However, available structures of complexes involving p53-TAD[70–73] show that TAD interacts with other proteins mainly via two short helices (see Fig 1A). The simple interaction or interface interruption mechanism thus cannot explain the effects of phosphorylation at several sites outside of the helical segments. Instead, the unbound state of p53-TAD must also be considered. Specifically, the disordered ensemble of free TAD is highly susceptible to post-translational modifications, which could alter the level of residual structures and modulate the entropy cost of folding upon specific binding to regulate the binding affinity. Such a mechanism has been demonstrated in our previous study of the CREB/CBP interaction[19]. The molecular mechanism of p53 activation by multisite phosphorylation is highly relevant for understanding how TAD cancer mutants may alter the spectrum of target gene transactivation[74] and contribute to the gradient of p53 tumor suppression function in cancers[75]. In particular, the current simulations strongly support that TAD cancer mutants can significantly modulate the unbound conformational ensembles, which could in turn disturb the balance between binding to MDM2 and CBP and further alter how the balance is regulated by multisite phosphorylation of TAD. Establishing the functional implications of the predicted cancer mutant modulation of the disordered ensembles will require additional experimental characterization of TAD cancer mutant structural properties as well as new biochemical and biophysical measurements of p53 binding thermodynamics with key regulatory proteins. The success of the current simulations demonstrates the feasibility and promise of combining advanced sampling techniques and modern atomistic force fields, particularly with implicit solvent, for effective IDP simulations. Coupled with appropriate structural and biophysical experiments, *de novo* atomistic simulations could provide a general framework for comparatively assessing the effects of disease-related mutations as well as post-translational modifications on IDP structure and interaction. At the same time, important limitations remain in implicit solvent protein force fields, and the simulated ensembles are overly compact. This has proven to be a key artifact that not only severely hinders our ability to generate highly converged ensembles but also greatly compromises reliable interpretation of predicted structural impacts of mutations. The current study thus also underpins the importance of continual development and optimization of implicit solvent protein force fields.

## Methods

### RE-GA implicit solvent simulations

Fully extended and helical conformations of the wild-type p53-TAD (residues 1–61: MEEPQ SDPSV EPPLS QETFS DLWKL LPENN VLSPLP SQAM DDLM LSPDDI EQWFT EDPGP D) were first generated using CHARMM [76,77]. Both termini were neutralized. These initial conformations were then used to initiate two independent RE-GA simulations (referred to as folding and control runs, respectively) in the GBSW/SA implicit solvent [78–80]. The GBSW/SA force field is based on the CHARMM22/CMAP protein force field [81–84], and has been previously optimized for simulation of conformational equilibria of small peptides. Despite several existing limitations [55,56], it has been reasonably successful in simulating the disordered ensembles of several IDPs [18–20] and unstable protein states [85–87]. The SHAKE algorithm [88] was applied to fix lengths of all hydrogen-involving bonds, and the dynamics time step was 2 fs. The nonbonded interactions were cut off at 16 Å, and the salt concentration was set to 0.1 M in GBSW. All RE-GA simulations were performed using the Multi-scale Modeling Tools in Structural Biology (MMTSB) Toolset [89] together with CHARMM. Each RE-GA run involved 16 replicas distributed exponentially between 300 and 500 K. Temperature exchanges were attempted every 2 ps. The replica occupying the lowest temperature was selected to undergo GA every 2000 RE cycles after the completion of the previous GA cycle [44]. The total length of all RE-GA simulations was 200 ns per replica, which proved sufficient for achieving excellent convergence in the calculated ensembles (see Results). The exchange acceptance ratios were about 25%. Additional 200-ns RE-GA simulations were initiated from fully extended conformations for five selected cancer-associated mutants of p53-TAD. E17D and K24N are frequently associated with female genital cancers [33]; D49Y and W53G are predicted to cause the largest changes in the disorder tendency based on metaPrDOS sequence analysis [90] and are associated with brain and bladder cancers, respectively [91,92]; and N29K/N30D is only complex cancer mutant known[33] and is associated with breast cancers [93].

### Structural, clustering and NMR analysis

Structural ensembles were constructed by collecting conformations sampled at 300 K during the RE-GA simulations. All subsequent structural and clustering analysis was performed using a combination of CHARMM, the MMTSB toolset and in-house scripts. Molecular visualization was generated using VMD [94]. For clustering analysis, the simulated ensembles were first under-sampled by only including snapshots sampled every 20 ps during the last 80 ns of each RE-GA simulations. The resulting 4000-member ensembles were clustered using the fixed radius clustering algorithm as implemented in the MMTSB/enscluster.pl tool (with—kclust option), with a cutoff radius of 5 Å Cα root-mean-square distance (RMSD).

Theoretical residual dipolar coupling (RDC) values were computed from the simulated ensembles using the PALES software [95], and the final ensemble-averaged RDC profiles were uniformly scaled to best reproduce the experimental data [39]. The theoretical paramagnetic relaxation enhancement (PRE) broadenings of several previously characterized sites of spin-label attachment (D7C, E28C, A39C and D61C)[37] were calculated for the wild-type p53-TAD. The theoretical ratios of ^1^H-^15^N HSQC peak intensities in the paramagnetic and diamagnetic samples were calculated as peak intensities in the paramagnetic sample, *I*_ox_ and the diamagnetic sample *I*_red_ were calculated theoretically using the equation $\frac{I_{\text{ox}}}{I_{\text{red}}}=\frac{R_{2}\text{ exp}(-R_{2}^{sp}t)}{R_{2}+R_{2}^{sp}}$ with $R_{2}^{sp}=\frac{K}{r^{6}}(4\tau_{C}+\frac{3\tau_{C}}{1+\omega_{H}^{2}\tau_{C}^{2}})$[96]. Here *r* is the ensemble-averaged residue-spin label distance, and *K* = 1.23×10^-32^ cm^6^s^-2^ for the interaction between a single electron and proton. The simulations did not include actual MTSL spin label used in NMR experiments [43]. Therefore, Cα-Cα distances were calculated to approximate the actual electron-proton separations. Consistent with the experimental work [43], Larmor frequency *ω*_H_ = 600 MHz, the average correlation time *τ*_*C*_ for the electron-nuclear dipole-dipole interaction is set to 3.3 ns, the average *R*_*2*_ relaxation time in absence of the paramagnetic center is set to 16 s^-1^, and the duration of the INEPT delay is set to *t* = 9.8 ms.

## Supporting Information

S1 Fig Self convergence of the structural ensembles of all five p53-TAD cancer mutants. (TIF)

S2 Fig Convergence of the distributions of helical substates of A) wild-type p53-TAD folding run, B) wild-type p53-TAD control run, C) p53-TAD K24N, and D) p53-TAD N29K/N30D. See Fig 7 caption for details of the contour plots. (TIF)

S3 Fig Centroids of the top four clusters from the last 80-ns of folding RE-GA simulation of the wild-type p53 TAD. A total of 315 clusters is identified in the 4000-member ensemble. The total populations of clusters of various size ranges (besides the top four clusters) are: 40–49: 6.9%, 30–39: 20.4%, 20–29: 25%, 10–19: 23.5%, and <10: 17.5%.
(TIF)

S4 Fig Centroids of the top four clusters from the last 80-ns of folding RE-GA simulation of p53 TAD K24N. A total of 313 clusters is identified in the 4000-member ensemble. The total populations of clusters of various size ranges (besides the top four clusters) are: 50–59: 3.6%, 40–49: 14.4%, 30–39: 14.9%, 20–29: 17.7%, 10–19: 27.5%, and <10: 14.7%.
(TIF)

S5 Fig Centroids of the top four clusters from the last 80-ns of folding RE-GA simulation of p53 TAD W53G. A total of 343 clusters is identified in the 4000-member ensemble. The total populations of clusters of various size ranges (besides the top four clusters) are: 50–79: 5.7%, 40–49: 4.1%, 30–39: 16.9%, 20–29: 21.9%, 10–19: 24.9%, and <10: 17.9%.
(TIF)

S6 Fig Centroids of the top four clusters from the last 80-ns of folding RE-GA simulation of p53 TAD D49Y. A total of 319 clusters is identified in the 4000-member ensemble. The total populations of clusters of various size ranges (besides the top four clusters) are: 50–59: 2.7%, 40–49: 13.5%, 30–39: 12.2%, 20–29: 23%, 10–19: 27%, and <10: 14.6%.
(TIF)

S7 Fig Centroids of the top three clusters from the last 80-ns of folding RE-GA simulation of p53 TAD E17D. A total of 328 clusters is identified in the 4000-member ensemble. The total populations of clusters of various size ranges (besides the top three clusters) are: 40–49: 17.5%, 30–39: 12.8%, 20–29: 19.2%, 10–19: 26.2%, and <10: 19%.
(TIF)

S8 Fig Centroids of the top four clusters from the last 80-ns of folding RE-GA simulation of p53 TAD N29K/N30D. A total of 312 clusters is identified in the 4000-member ensemble. The total populations of clusters of various size ranges (besides the top four clusters) are: >50: 7.5%, 40–49: 15.2%, 30–39: 9.4%, 20–29: 19.8%, 10–19: 24%, and <10: 16.9%.
(TIF)
