# Supplementary figures and images for: Modulation of the Disordered Conformational Ensembles of the p53 Transactivation Domain by Cancer-Associated Mutations
Source: PLoS Comput Biol. 2015 Apr 21;11(4):e1004247. doi: 10.1371/journal.pcbi.1004247 (PMC4405366; doi:10.1371/journal.pcbi.1004247)

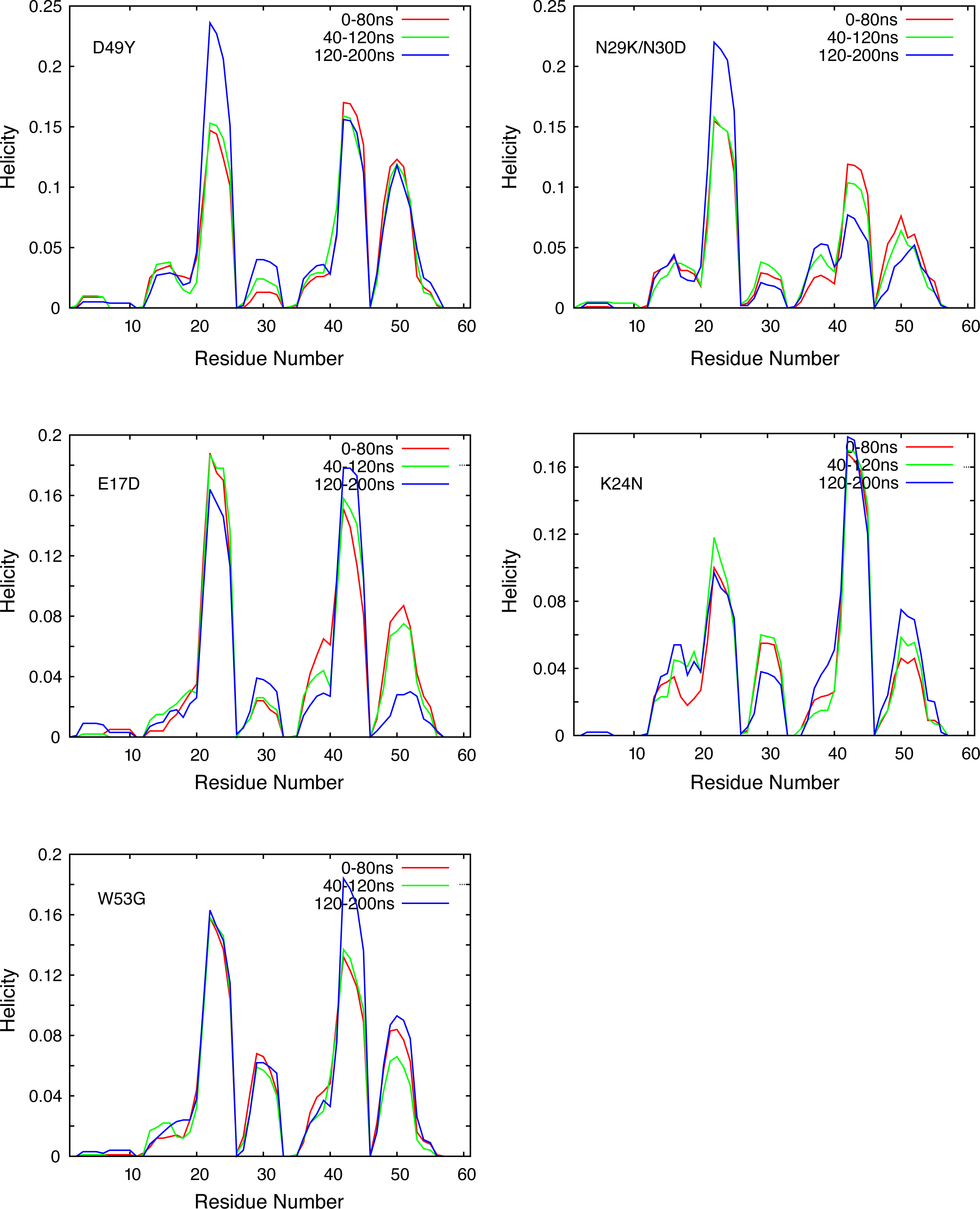

Supplement: S1 Fig — (TIF) [file pcbi.1004247.s001.tif]

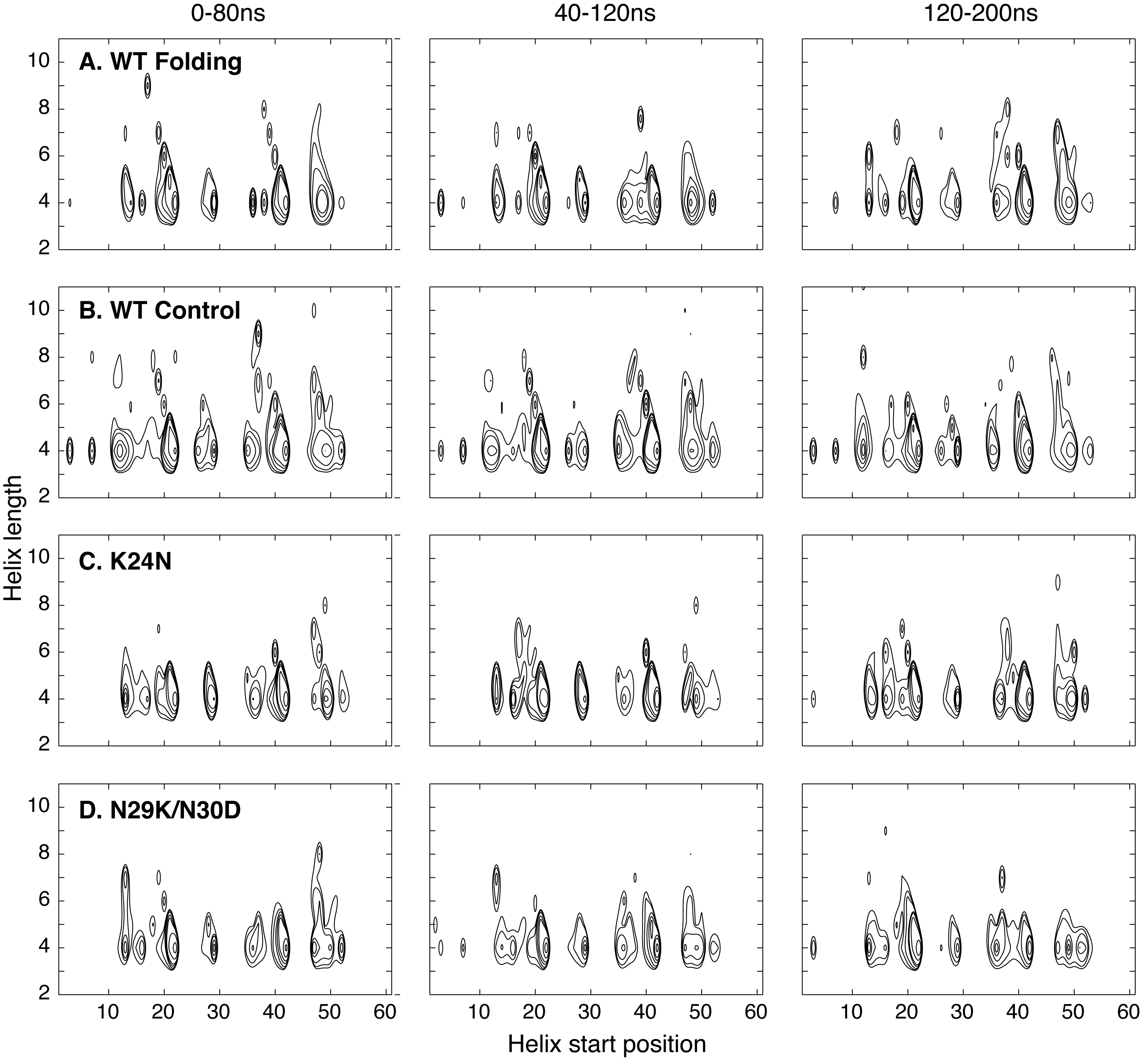

Supplement: S2 Fig — (TIF) [file pcbi.1004247.s002.tif]

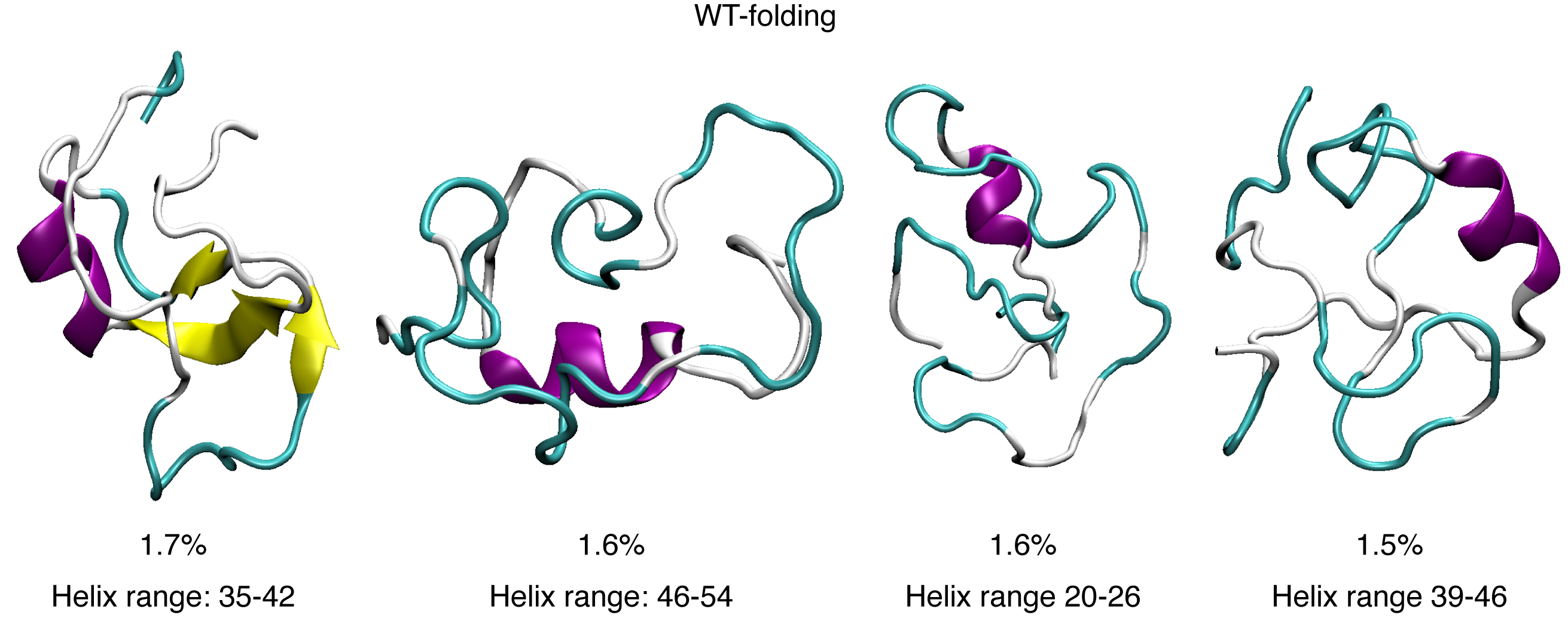

Supplement: S3 Fig — A total of 315 clusters is identified in the 4000-member ensemble. The total populations of clusters of various size ranges (besides the top four clusters) are: 40–49: 6.9%, 30–39: 20.4%, 20–29: 25%, 10–19: 23.5%, and <10: 17.5%. (TIF) [file pcbi.1004247.s003.tif]

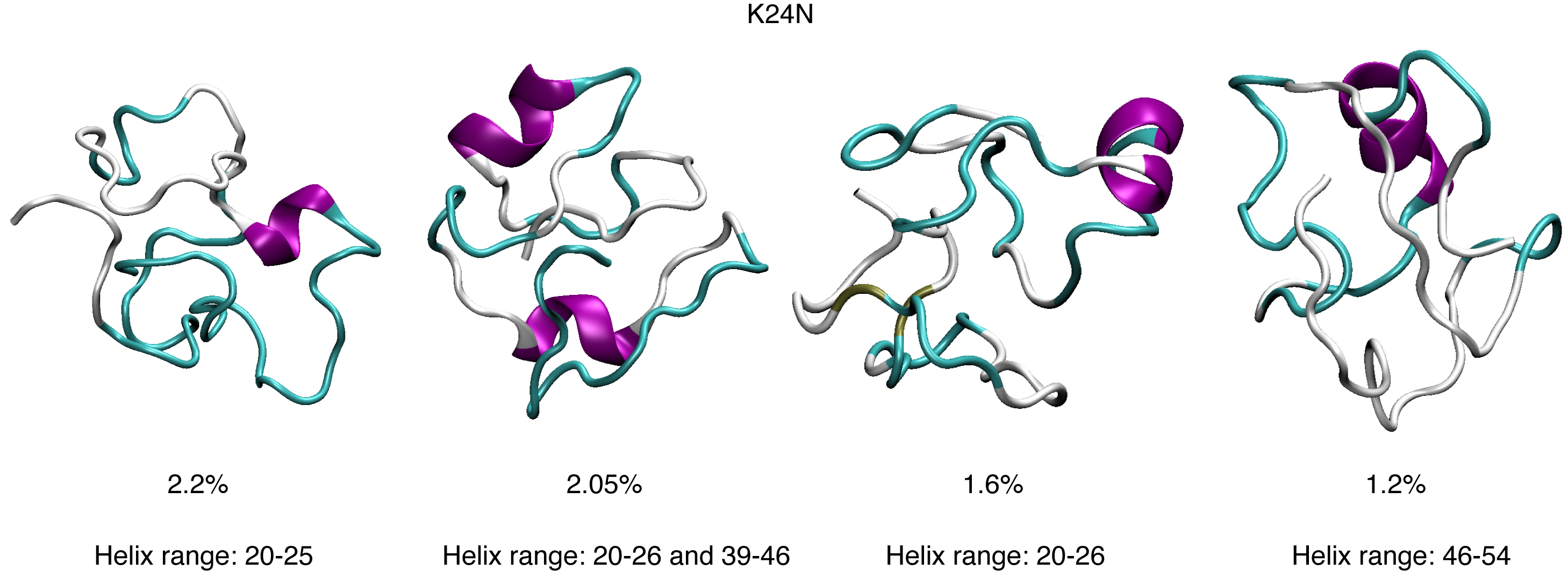

Supplement: S4 Fig — A total of 313 clusters is identified in the 4000-member ensemble. The total populations of clusters of various size ranges (besides the top four clusters) are: 50–59: 3.6%, 40–49: 14.4%, 30–39: 14.9%, 20–29: 17.7%, 10–19: 27.5%, and <10: 14.7%. (TIF) [file pcbi.1004247.s004.tif]

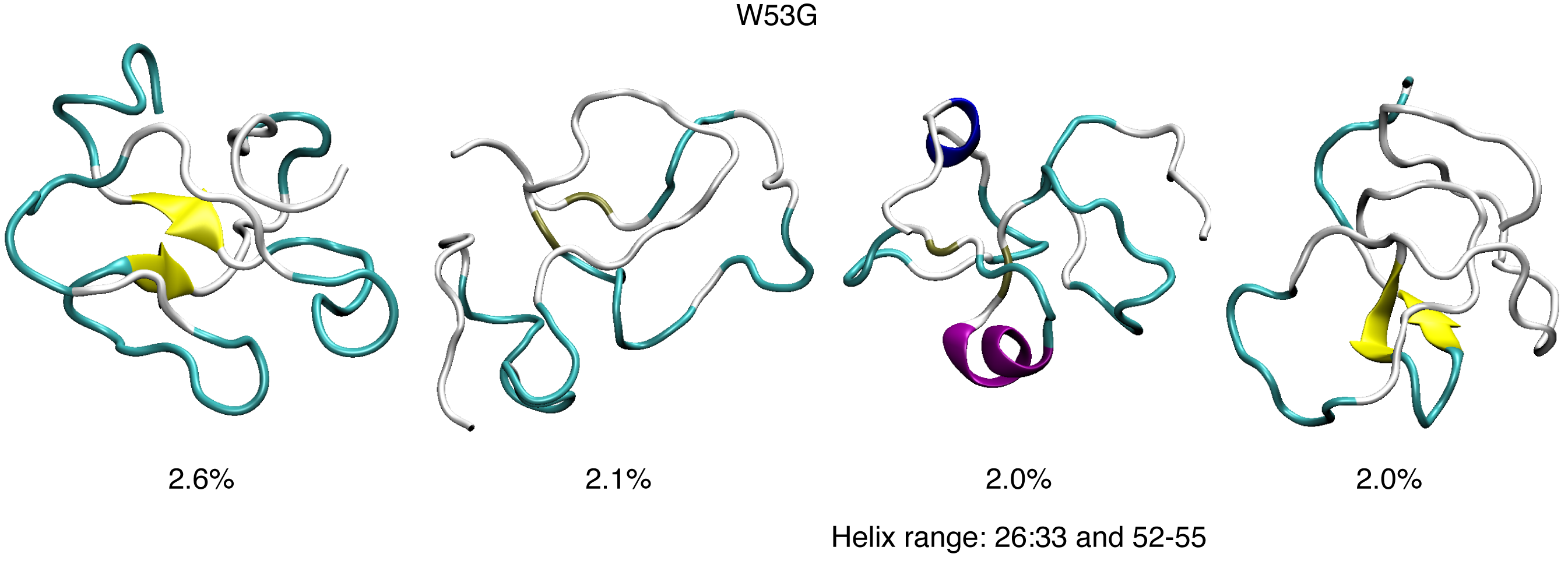

Supplement: S5 Fig — A total of 343 clusters is identified in the 4000-member ensemble. The total populations of clusters of various size ranges (besides the top four clusters) are: 50–79: 5.7%, 40–49: 4.1%, 30–39: 16.9%, 20–29: 21.9%, 10–19: 24.9%, and <10: 17.9%. (TIF) [file pcbi.1004247.s005.tif]

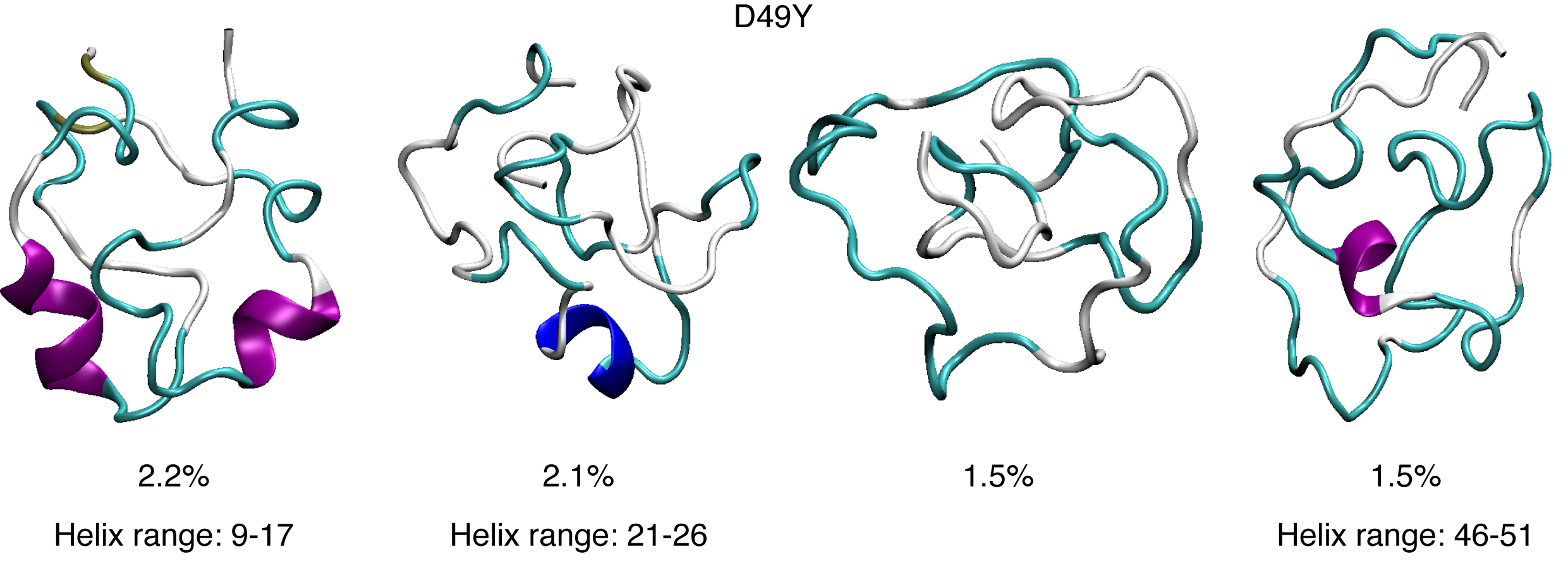

Supplement: S6 Fig — A total of 319 clusters is identified in the 4000-member ensemble. The total populations of clusters of various size ranges (besides the top four clusters) are: 50–59: 2.7%, 40–49: 13.5%, 30–39: 12.2%, 20–29: 23%, 10–19: 27%, and <10: 14.6%. (TIF) [file pcbi.1004247.s006.tif]

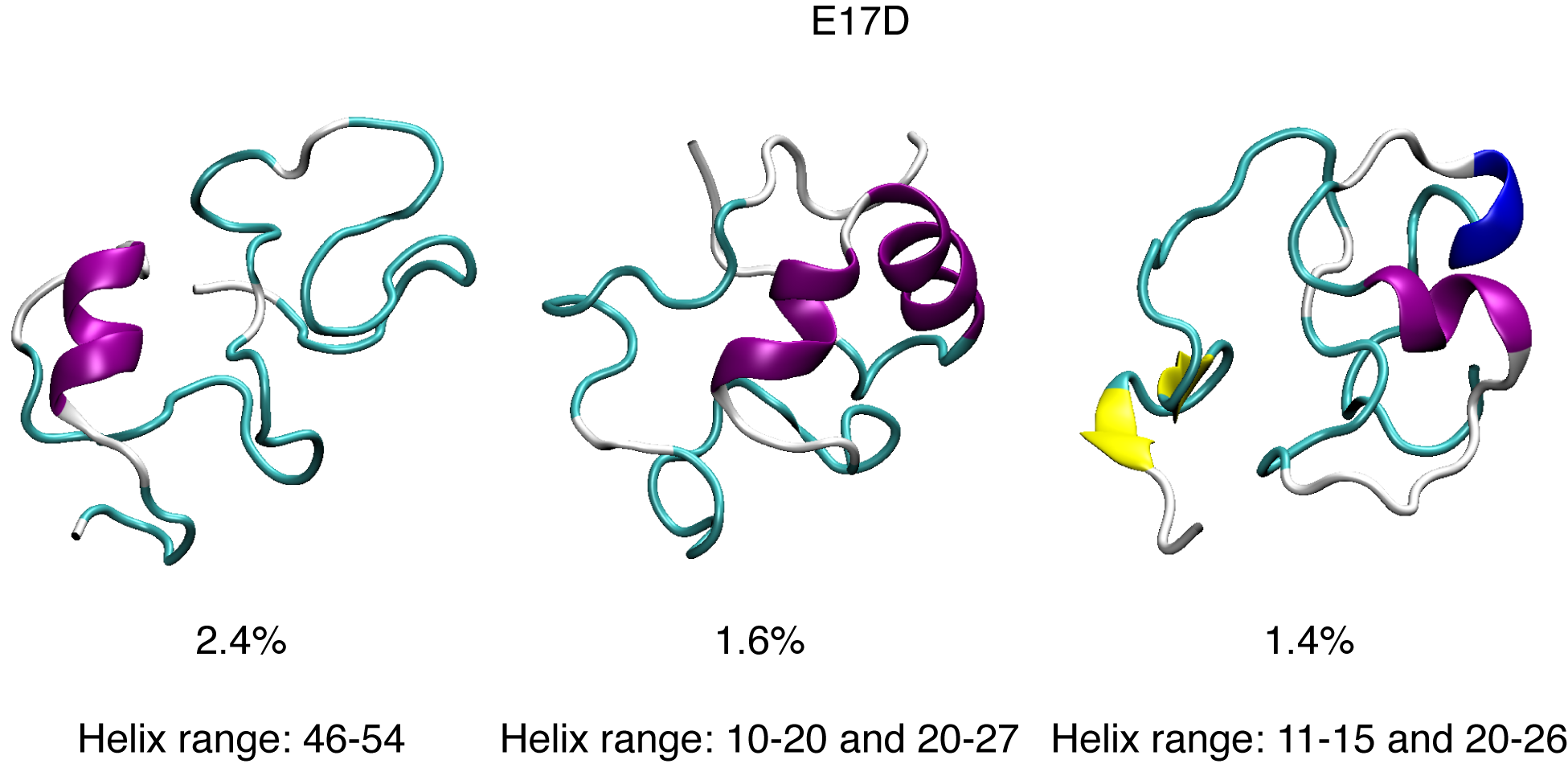

Supplement: S7 Fig — A total of 328 clusters is identified in the 4000-member ensemble. The total populations of clusters of various size ranges (besides the top three clusters) are: 40–49: 17.5%, 30–39: 12.8%, 20–29: 19.2%, 10–19: 26.2%, and <10: 19%. (TIF) [file pcbi.1004247.s007.tif]

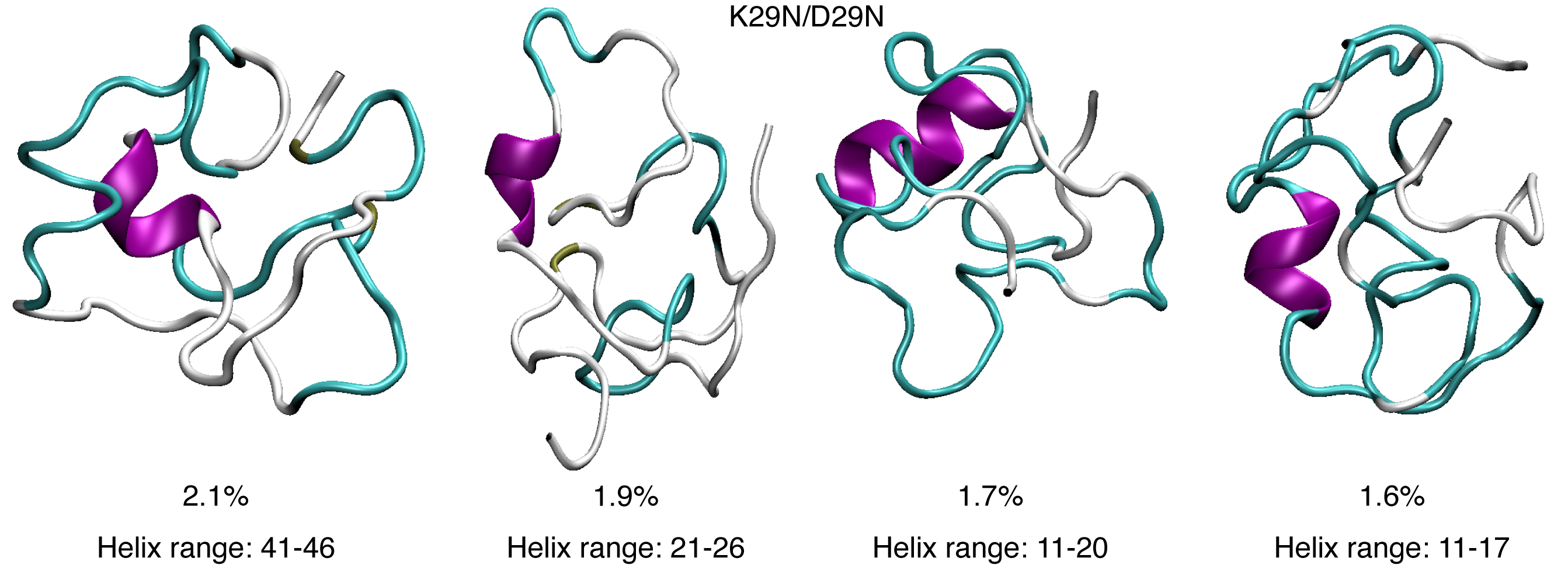

Supplement: S8 Fig — A total of 312 clusters is identified in the 4000-member ensemble. The total populations of clusters of various size ranges (besides the top four clusters) are: >50: 7.5%, 40–49: 15.2%, 30–39: 9.4%, 20–29: 19.8%, 10–19: 24%, and <10: 16.9%. (TIF) [file pcbi.1004247.s008.tif]
